# Comparison of ocular changes in multiple sclerosis and neuromyelitis optica spectrum disorder patients

**DOI:** 10.3389/fneur.2024.1417814

**Published:** 2024-08-19

**Authors:** Xiaoyue Wang, Li Bao

**Affiliations:** Department of Ophthalmology, West China Hospital, Sichuan University, Chengdu, Sichuan, China

**Keywords:** multiple sclerosis, neuromyelitis optica spectrum disorder, spectral domain optical coherence tomography, visual evoked potential, ocular changes

## Abstract

**Purpose:**

To explore ocular changes in patients with MS and NMOSD via SD-OCT and PVEP analysis.

**Methods:**

From August 2020 to July 2021, 82 patients (164 eyes) diagnosed with MS, 59 patients (118 eyes) diagnosed with NMOSD and 50 healthy controls (100 eyes) were retrospectively selected. SD-OCT and PVEP were performed to compare retinal nerve fibre layer (RNFL) thickness around the optic disc, ganglion cell inner plexiform layer (GCIPL) thickness in the macula and P100 latency and amplitude between the disease groups and the control group.

**Results:**

In the NMOSD and MS groups, the thickness of the GCIPL quadrants in eyes with optic neuritis was thinner than that in eyes without optic neuritis, and the amplitude of the P100 wave decreased. In addition, in eyes with optic neuritis, patients with NMOSD have thinner RNFL thicknesses in the temporal and superior quadrants than patients with MS, and the thickness of the GCIPL is thinner in each region. In eyes without optic neuritis, patients with MS have thinner nasal RNFL than do those with NMOSD.

**Conclusion:**

SD-OCT and VEP may be useful for monitoring and distinguishing pathological changes in MS and NMOSD patients.

## Background

Multiple sclerosis (MS) is a representative inflammatory demyelinating disease of the central nervous system that often affects young people aged 20–40 years. Typical MS is characterized by affliction in multiple parts of the body at different times throughout the patient’s life, and the course of disease mostly involves relapse, remission, and ladder-like aggravation. Neuromyelitis optica spectrum disorder (NMOSD) is a common acute or subacute demyelinating disease of the central nervous system in which the optic nerve and the spinal cord are involved simultaneously or successively; however, the brain is rarely affected. Most NMOSDs are recurrent but rarely progress to secondary progression, and they have a significantly greater recurrence frequency than MS does. The two diseases themselves usually do not threaten the lives of patients, but the high disability rate caused by repeated attacks causes great harm to their physical and mental health.

The exact pathophysiological mechanisms underlying MS and NMOSD have not been fully elucidated; however, there is evidence that tissue damage and demyelination in MS are mediated by T-cell activity and that axonal and neuronal atrophy may be secondary effects of inflammatory demyelination but may also be the result of independent subclinical disease activity (1). Neuraxial degeneration (in addition to demyelination) has recently been thought to be more relevant to MS pathophysiology, and it has been documented in both active and inactive lesions, distal to areas affected by autoimmune inflammation, and early in the disease process (2). In contrast, the pathophysiology of NMOSD primarily involves the deposition of IgG and complement, resulting in the loss of the AQP4 protein on astrocytes and severe neuronal and axonal loss (3). However, in both disorders, visual impairment appears to be common, and acute optic neuritis (ON) often occurs as the initial symptom (4, 5). ON is an inflammatory reaction of the optic nerve that is often accompanied by pain during eye movements, followed by vision loss. Recovery of visual function is often incomplete and may be due to ongoing demyelination or, in more severe cases, axonal loss (6). Compared with MS, ON in NMOSD is generally more severe, more recurrent, and often bilateral (7, 8). In addition, there are many differences in the treatment of patients with MS and NMOSD, and if not treated properly, repeated episodes of the disease can accelerate the impairment of neurological function. Therefore, distinguishing between MS and NMOSD at an early stage is particularly important. At present, the differential diagnosis of these two diseases is performed mainly by AQP4-IgG antibody detection, but approximately 25% of patients do not have anti-AQP4 antibodies but may have anti-MOG (MOGAD) or may be simply seronegative.

Spectral domain optical coherence tomography (SD-OCT) is a safe and noninvasive 3D imaging tool that uses low-coherence near-infrared light to generate a cross-sectional image of the retina that can be used to quantify axonal and neuronal atrophy (9). Visual evoked potential (VEP) is a neurophysiological examination of the subcortical structure of the visual ascending pathway. Electrical activity is generated in 17 areas of the visual cortex of the occipital lobe of the brain after the retina is stimulated by a flash of light or an image. The examination is highly sensitive, stable, and repeatable, which makes it a reliable and objective examination method and an important means for clinically evaluating optic neuropathy.

In this study, VEP and SD-OCT were used to comprehensively observe the changes in the retina and optic nerve in patients with MS and NMOSD from structural and functional perspectives to determine the differences between the two diseases in terms of retinal and optic nerve changes and to provide an objective basis for the early diagnosis and treatment of these two diseases and for monitoring disease progression.

## Materials and methods

### Materials

This was a retrospective clinical observational study. This research was approved by the ethics committee of West China Hospital, Sichuan University (NO. 749 of 2020), and all patients were notified in advance and signed a written informed consent form. The authors had access to information that could identify individual participants during or after data collection.

From Aug 2020 to July 2021, 82 patients (164 eyes) diagnosed with multiple sclerosis at West China Hospital of Sichuan University were selected as the MS group, 59 patients (118 eyes) diagnosed with optic neuromyelitis spectrum disorder were selected as the NMOSD group, and 50 healthy volunteers (100 eyes) were selected as the control group. Patients in both the MS and NMOSD groups had a disease duration of more than 2 years and had no active ON 6 months prior to enrolment. The MS and NMOSD groups were divided into MS−ON, MS + ON, NMOSD−ON and NMOSD+ON groups according to their history of ON. The sex and age of the individuals in the groups are shown in Table 1.

**Table 1:** Basic information of the research subjects.

	NMOSD group			MS group			Control group	*F* value	*p* value
	+ON	−ON	SUM	+ON	−ON	SUM			
Number of eyes enrolled	62	56	118	66	98	164	100	NA	NA
Sex (female/male)	28/8	17/6	45/14	26/18	23/15	49/33	36/14	5.210	0.266
Age (years, mean ± SD)	46.33 ± 12.87	49.02 ± 13.58	48.17 ± 14.94^#*^	39.68 ± 11.45	35.59 ± 9.39	35.45 ± 10.87	40.26 ± 12.38	19.595	0.008
Disease duration (years, median, range)	3.6(2.2–8)	2.8(2–7)	3 (2–8)	3.5(3–8)	4.5(3–9)	4 (3–9)	NA	NA	NA
Long-term treatment									
Receptor modulator	38			69			NA	NA	NA
Biotherapy	21			13			NA	NA	NA

^#^Compared with MS group, *p* < 0.05;*compared with Control group, *p* < 0.05; ^&^compared with NMOSD-ON group, *p* < 0.05; ^@^compared with NMOSD-ON group, ^compared with NMOSD-ON group, ^$^compared with NMOSD-ON group. NA, not applicable; NMOSD, neuromyelitis optica spectrum disorder; MS, multiple Sclerosis; ON, optic neuritis.

#### Inclusion criteria

The MS diagnosis was determined according to the 2017 revised McDonald MS diagnostic standard (10). The inclusion criterion was relapsing–remitting. The NMOSD diagnosis was determined according to the diagnostic standard of optic neuromyelitis pedigrees revised by Wingerchuk et al. in 2015 (11), and all patients were AQP4 positive. The course of the disease in all patients should be no less than 2 years from the time of initial diagnosis to the time of testing. All MS patients and NMOSD patients were treated with pulse glucocorticoids during the acute exacerbation period, and 20 NMOSD patients also underwent plasmapheresis therapy. After the acute stage, different drugs were selected for sequential treatment during the remission period.

#### Exclusion criteria

**Figure 1:**
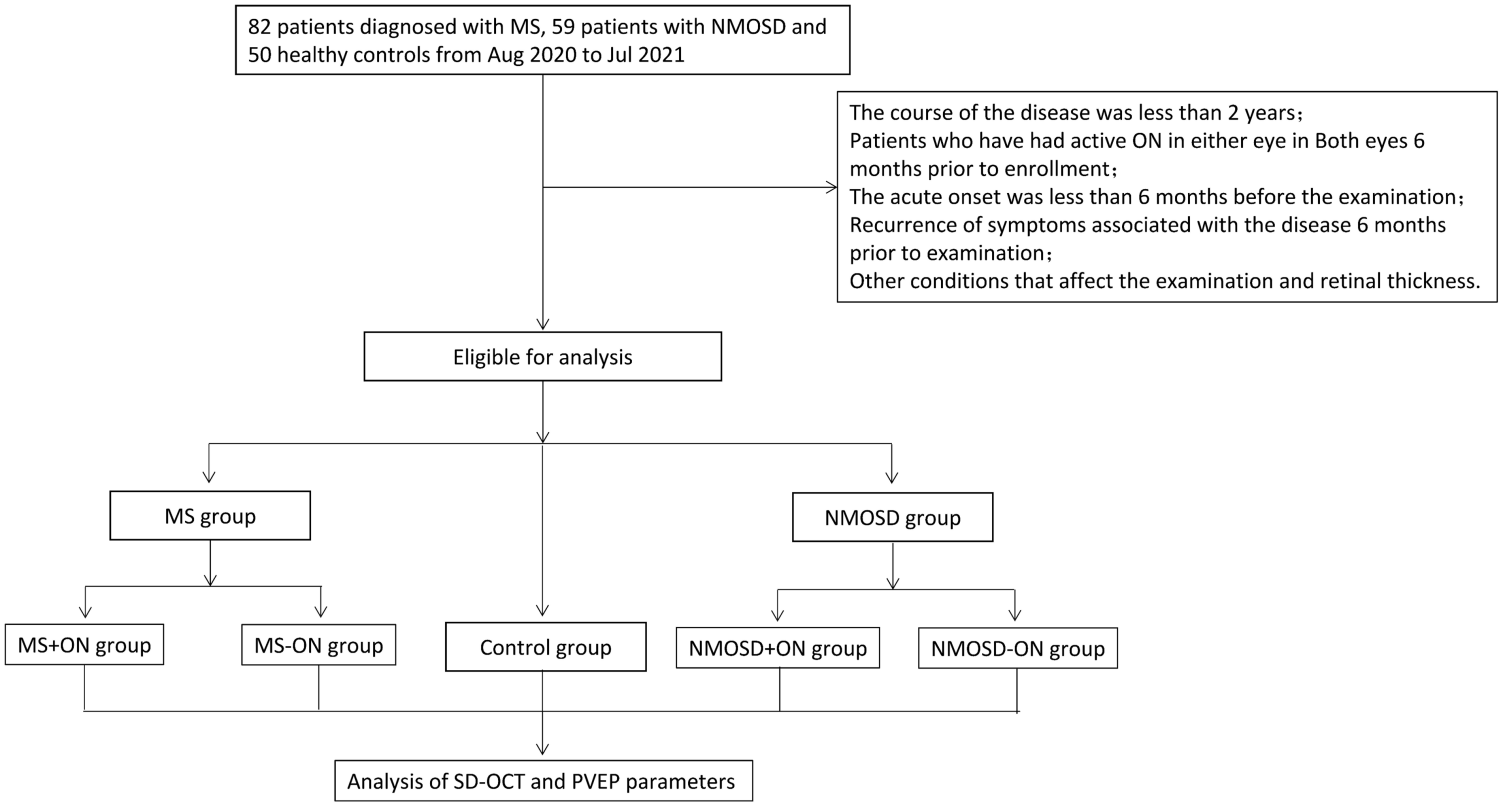
The flowchart of the study shows the inclusion and exclusion criteria of the study as well as the grouping and study pathways of the studies.

The course of the disease was less than 2 years; patients who had active ON in either eye in both eyes 6 months prior to enrolment; acute onset was less than 6 months before the examination; recurrence of symptoms associated with the disease 6 months prior to examination; subclinical optic neuritis; equivalent spherical degree>| ± 3D|; intraocular pressure > 21 mmHg; obvious opacity of the refractive stroma affects imaging; an aphakic study eye; glaucoma or high intraocular pressure; pathological myopia, diabetic retinopathy, retinal vein occlusion, proliferative vitreoretinopathy, macular holes, a macular epiretinal membrane, idiopathic or autoimmune uveitis or other fundus diseases; scleral malacia in any eye; active eye infection in any eye; renal insufficiency and serious cardiovascular and cerebrovascular diseases; and recent preparations for childbirth, pregnancy or lactation (shown in Figure 1).

### Inspection method

#### Best corrected visual acuity (BCVA)

The examination was carried out in a relatively dark room. The subjects were optometrically tested with a retinoscope and a comprehensive refractometer, and then the BCVA was determined with a standard logarithmic visual acuity chart and is expressed as the logarithm of the minimum angle of the resolution value. For values greater than 1, the standard value was used; for example, visual acuity was 1.7 for counting fingers, 2.0 for manual recording and 2.6 for light perception (12).

#### Visual-evoked potential (VEP)

A Roland visual electrophysiological examination system (Germany) was adopted, and a Ag-Ag-Cl skin electrode was used for recording. The electrode placement was in accordance with international ISCEV standards (13). Each test was administered an average of ≥64 times, and the average of each spatial frequency test was taken twice for each eye. All patients were given priority for pattern visual-evoked potential (PVEP) examination to analyse the latency and amplitude of the P100 wave.

#### Spectral domain optical coherence tomography (SD-OCT)

**Figure 2:**
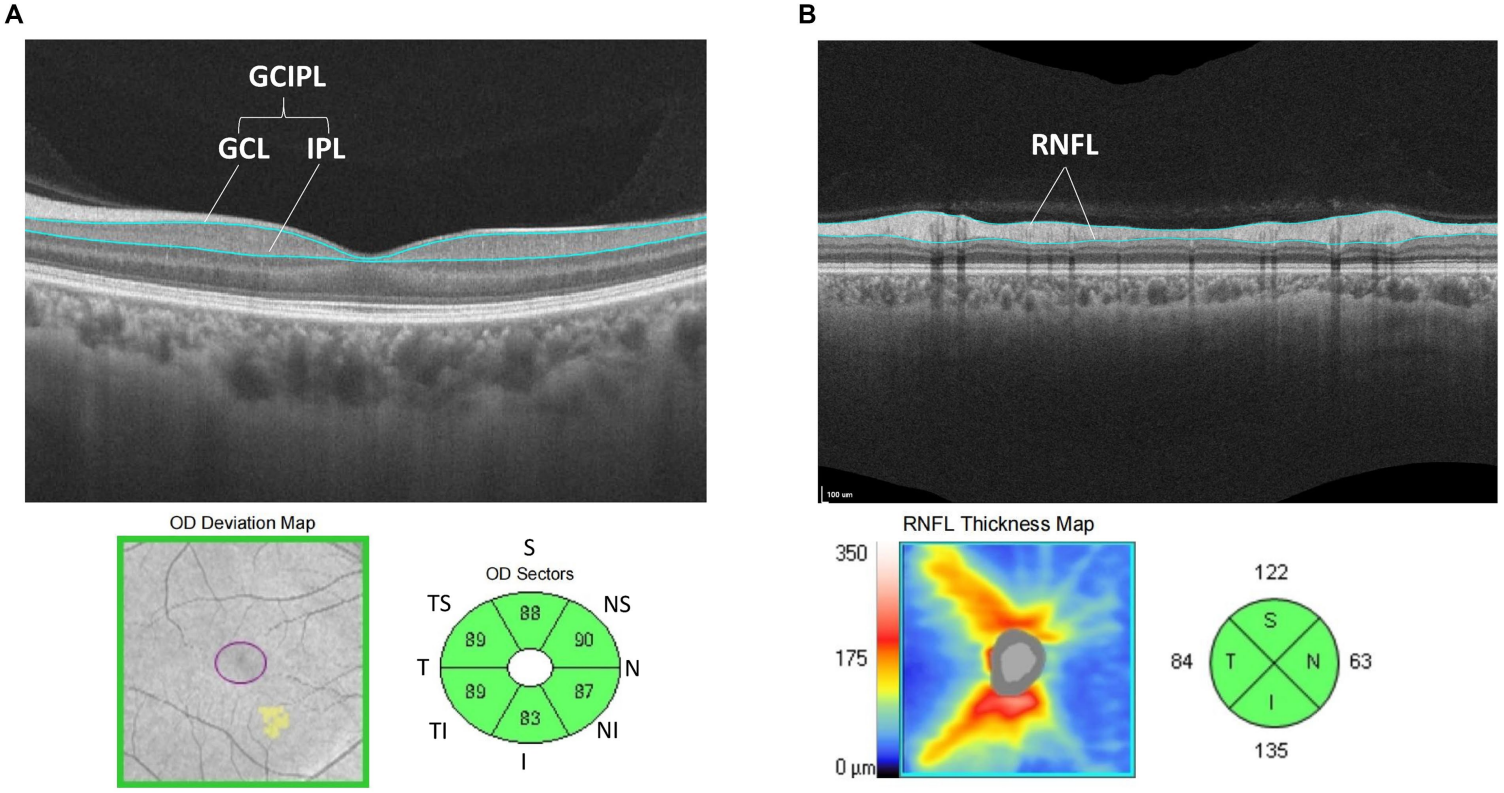
**(A)** Schematic diagram of GCIPL thickness measurement: analyze the average thickness of GCL to IPL in the area of the macula was with an ellipse ring (horizontal diameter of 4 mm and vertical diameter of 4.8 mm). Divide this area into six directions: superior, temple superior, temple inferior, nasal superior, nasal inferior and inferior area. **(B)** Schematic diagram of RNFL thickness measurement: analysis the average thickness of RNFL in the area of the optic disc with a ring diameter of 3.46 mm and was divided into four quadrants: superior, temporal, nasal, and inferior.

A CIRRUS MODEL 5000 HD-OCT scanner (Carl Zeiss, Germany) was used. The image processing of OCT was carried out automatically (SW Ver: 11.1.0.32456, Copyright 2018, Carl Zeiss Meditec). The macular cube 512*128 mode was used to scan the retina with the macular fovea as the origin and a diameter range of 6 mm, and ganglion cell OU analysis was used to analyse the average ganglion cell–inner plexiform layer thickness (GCIPL) in the area of the macula with an ellipse ring (horizontal diameter of 4 mm and vertical diameter of 4.8 mm), which was divided into six quadrants: superior, inferior, temporal superior, temporal inferior, nasal superior and nasal inferior (see Figure 2A).

Then, the Optic Disc Cube 200*200 mode was used to scan the retina with a diameter of 3.46 mm in both eyes, with the centre of the optic disc as the origin, and the average nerve fibre layer (RNFL) thickness analysis of the optic disc revealed a ring diameter of 3.46 mm, which was divided into four quadrants: superior, temporal, nasal, and inferior (see Figure 2B). A signal strength >8 was considered credible. The OSCAR-IB criteria were used to check for sufficient quality of SD-OCT examinations, following the APOSTEl 2.0 recommendations for OCT data.

### Statistical methods

Statistical analysis was performed via SPSS 23.0 (SPSS, Inc., Chicago, USA). The single-sample Kolmogorov–Smirnov test was used for all the study data, and the data in this study conformed to a normal distribution. The concentration trend of the normally distributed sizable data was described by the mean ± standard deviation (x ± SD), the *t* test was used for comparisons of two independent samples, and analysis of variance was used for comparisons of multiple samples. The chi-square test was used to compare differences between sexes; one-way ANOVA was used to compare the differences in age and OCT and VEP parameters between the MS group, NMOSD group and control group; and the Bonferroni correction was used for comparisons between the two groups. Independent samples t tests were used to compare the differences in the OCT and VEP parameters between the MS + ON and MS-ON groups and between the NMOSD+ON and NMOSD−ON groups. The area under the receiver operating characteristic (ROC) curve (AUC) was used to calculate the diagnostic power of the selected parameters in differentiating between NMOSD and MS eyes. Statistical significance was established at *p* < 0.05.

## Results

Basic information: NMOSD patients were older than MS patients and control group, and there were no differences in age or sex amongst the other subgroups. The detailed data are shown in Table 1.

### Comparison of BCVA differences amongst groups

**Figure 3:**
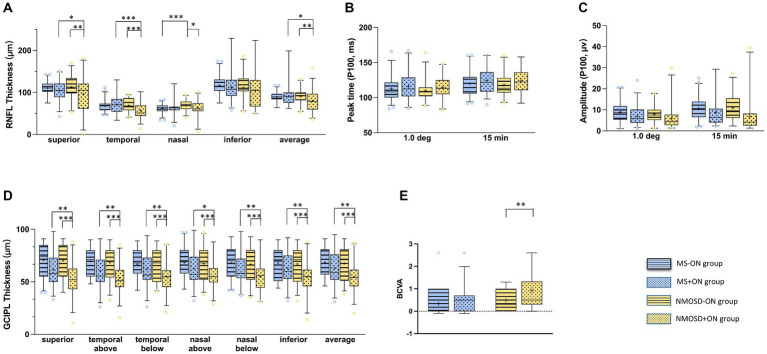
Comparison of the mean (+) of each parameter in MS-ON and its group (blue with strip), MS + ON and its group (blue with dots), NMOSD-ON and its groups (yellow with strip), NMOSD+ON and its groups (yellow with dots): **(A)** average retinal nerve fibre layer thickness, **(B)** peak time of P100 wave in PVEP, **(C)** amplitude of P100 wave at in PVEP, **(D)** average ganglion cell inner plexiform layer thickness,**(E)** best corrected visual acuity. The box and whisker plots show the 2.5–97.5 percentile number in each group. The blue circles (MS and its groups) and yellow circles (NMOSD and its groups) represented the values beyond the range of the 2.5–97.5 percentile. *p* < 0.001was marked with ***, 0.001 ≤ *p* < 0.01 was marked with **, 0.01 ≤ *p* < 0.05 was marked with *.

Differences in BCVA between groups were compared. In the −ON eyes, the BCVAs of the MS and NMOSD groups were lower than that of the control group (*p* < 0.05), and the BCVAs of the +ON patients were significantly lower than those of the −ON patients in the NMOSD group (*p* < 0.01, shown in Figure 3). The detailed data are shown in Table 2.

**Table 2:** BCVA, PVEP and OCT Datas of Multiple Sclerosis and neuromyelitis optica spactrum disorder patients with or without optic neuritis (Mean ± SD).

	MS group				NMOSD Group				Control Group
	MS−ON	MS + ON	*t*	*p*	NMOSD−ON	NMOSD+ON	*t*	*p*	
BCVA (LogMAR)	0.33 ± 0.49^&^	0.46 ± 0.48^*^	−1.706	0.090	0.49 ± 0.51^&^	0.91 ± 0.92	−3.092	0.002	−0.01 ± 0.03
RNFL Thickness (um)									
Superior	110.54 ± 16.03^&^	110.70 ± 45.73^*^	−0.031	0.975	112.27 ± 25.50^&^	95.81 ± 38.99	2.683	0.008	124.60 ± 18.18
Temporal	68.04 ± 12.87^&^	71.88 ± 22.01^*^	−1.407	0.161	68.80 ± 15.58^&^	57.10 ± 17.79	3.784	<0.001	85.02 ± 17.05
Nasal	62.93 ± 10.07^#&^	64.61 ± 15.15	−0.852	0.395	69.20 ± 11.16^&^	63.58 ± 15.34	2.253	0.026	69.58 ± 11.71
Inferior	116.38 ± 23.21^&^	113.55 ± 33.05	−0.645	0.520	111.34 ± 31.29^&^	104.45 ± 40.97	1.018	0.311	131.33 ± 21.52
Average	89.53 ± 11.09^&^	90.70 ± 23.35^*^	−0.428	0.669	90.91 ± 16.99^&^	79.94 ± 23.21	2.904	0.004	102.88 ± 10.27
GCIPL Thickness (um)									
Superior	70.91 ± 15.48^&^	61.74 ± 15.90^*^	3.679	<0.001	70.04 ± 15.70^&^	53.53 ± 15.21	5.800	<0.001	82.94 ± 8.13
Temporal superior	69.44 ± 13.96^&^	60.35 ± 14.99^*^	3.968	<0.001	68.18 ± 14.63^&^	53.73 ± 13.17	5.647	<0.001	81.59 ± 7.81
Temporal inferior	66.79 ± 15.51^&^	62.33 ± 15.08^*^	1.823	0.070	66.20 ± 15.62^&^	54.87 ± 14.09	4.140	<0.001	81.47 ± 9.31
Nasal superior	68.33 ± 16.29^&^	62.55 ± 15.37^*^	2.279	0.024	67.27 ± 16.03^&^	56.44 ± 13.96	3.923	<0.001	84.16 ± 8.95
Nasal inferior	68.01 ± 15.22^&^	63.02 ± 14.93^*^	2.077	0.039	66.61 ± 15.61^&^	55.08 ± 14.36	4.177	<0.001	82.06 ± 7.62
Inferior	68.03 ± 15.08^&^	62.94 ± 14.08^*^	2.177	0.031	67.02 ± 15.19^&^	54.87 ± 15.15	4.345	<0.001	78.89 ± 8.12
Average	68.58 ± 15.07^&^	62.59 ± 14.86^*^	2.510	0.013	67.54 ± 15.14^&^	54.84 ± 13.97	4.737	<0.001	92.10 ± 10.06
PVEP									
peak time (1.0 deg) (ms)	112.70 ± 16.88^&^	116.22 ± 18.45	−1.263	0.208	109.73 ± 12.33^&^	114.60 ± 15.56	−1.868	0.064	93.39 ± 11.12
amplitude (1.0 deg)(uv)	8.95 ± 4.87^&^	6.92 ± 4.13	2.781	0.006	7.99 ± 4.12^&^	5.94 ± 5.21	2.363	0.020	24.67 ± 12.23
peak time (15 min) (ms)	119.90 ± 17.55^&^	121.33 ± 26.22	−0.420	0.675	118.10 ± 14.38^&^	123.93 ± 17.65	−1.956	0.053	95.98 ± 13.36
amplitude (15 min) (uv)	10.63 ± 5.09^&^	8.78 ± 6.82	1.988	0.048	10.94 ± 6.50^&^	6.74 ± 7.27	3.296	0.001	27.27 ± 14.52

^#^Compared with NMOSD-ON group, *p* < 0.05; ^*^compared with NMOSD + ON group, *p* < 0.05; ^&^compared with Control group, *p* < 0.05. NMOSD, neuromyelitis optica spectrum disorder; MS, multiple Sclerosis; ON, optic neuritis; BCVA, best corrected visual acuity; RNFL, etinal nerve fibre layer; GCIPL, ganglion cell-inner plexiform layer; PVEP, pattern reveasal visual evoked potential.

### Comparison of the differences in the thickness of the peripapillary nerve fibre layer amongst the groups

The differences in RNFL thickness between the groups were compared. In the -ON eyes, the RNFL in all quadrants centred on the optic disc in the MS and NMOSD groups was thinner than that in the control group (*p* < 0.05), whereas the nasal quadrant RNFL thickness in the MS group was thinner than that in the NMOSD group (*p* < 0.05). In the +ON eyes, the RNFL thickness in the superior and temporal quadrants was lower in the NMOSD group than in the MS group (*p* < 0.05). Intragroup comparisons revealed that in the NMOSD group, the RNFL thickness in the nasal and temporal quadrants was lower in the +ON eyes than in the −ON eyes (*p* < 0.05), whereas there was no difference between the +ON eyes and the −ON eyes in the MS group (*p* > 0.05) (The detailed data are shown in Figure 3 and Table 2).

### Comparison of differences in GCIPL thickness in the macular region amongst the groups

The differences in GCIPL thickness between the groups were compared. In the −ON eyes, the thickness of the GCIPL in all regions centred on the fovea of the macula in the MS and NMOSD groups was lower than that in the control group (*p* < 0.05), but there was no difference between the MS group and the NMOSD group (*p* > 0.05). In the +ON eyes, the thickness of the GCIPL in all regions was lower in the NMOSD group than in the MS group (*p* < 0.05). The comparison between subgroups revealed that the thickness of the GCIPL in all regions of +ON eyes was thinner than that of −ON eyes in the NMOSD group and in the MS group, except for the temporal region (*p* < 0.05) (The detailed data are shown in Figure 3 and Table 2).

### Comparison of the PVEP P100 wave parameters amongst the groups

The differences between the peak time and amplitude of the PVEP P100 wave were compared between the groups. Compared with the control eyes, the MS group and the NMOSD group presented a delayed peak time and decreased amplitude of P100 waves (*p* < 0.05), whereas there was no difference between the MS and NMOSD groups (*p* > 0.05). In the +ON eyes, there was no difference between the MS and NMOSD groups. A comparison between subgroups revealed that the amplitude of P100 waves in +ON eyes was lower than that in -ON eyes at the 1° and 15′ spatial frequencies in the MS group and NMOSD group (*p* < 0.05) (The detailed data are shown in Figure 3 and Table 2).

### Diagnostic accuracy of the SD-OCT parameters

**Figure 4:**
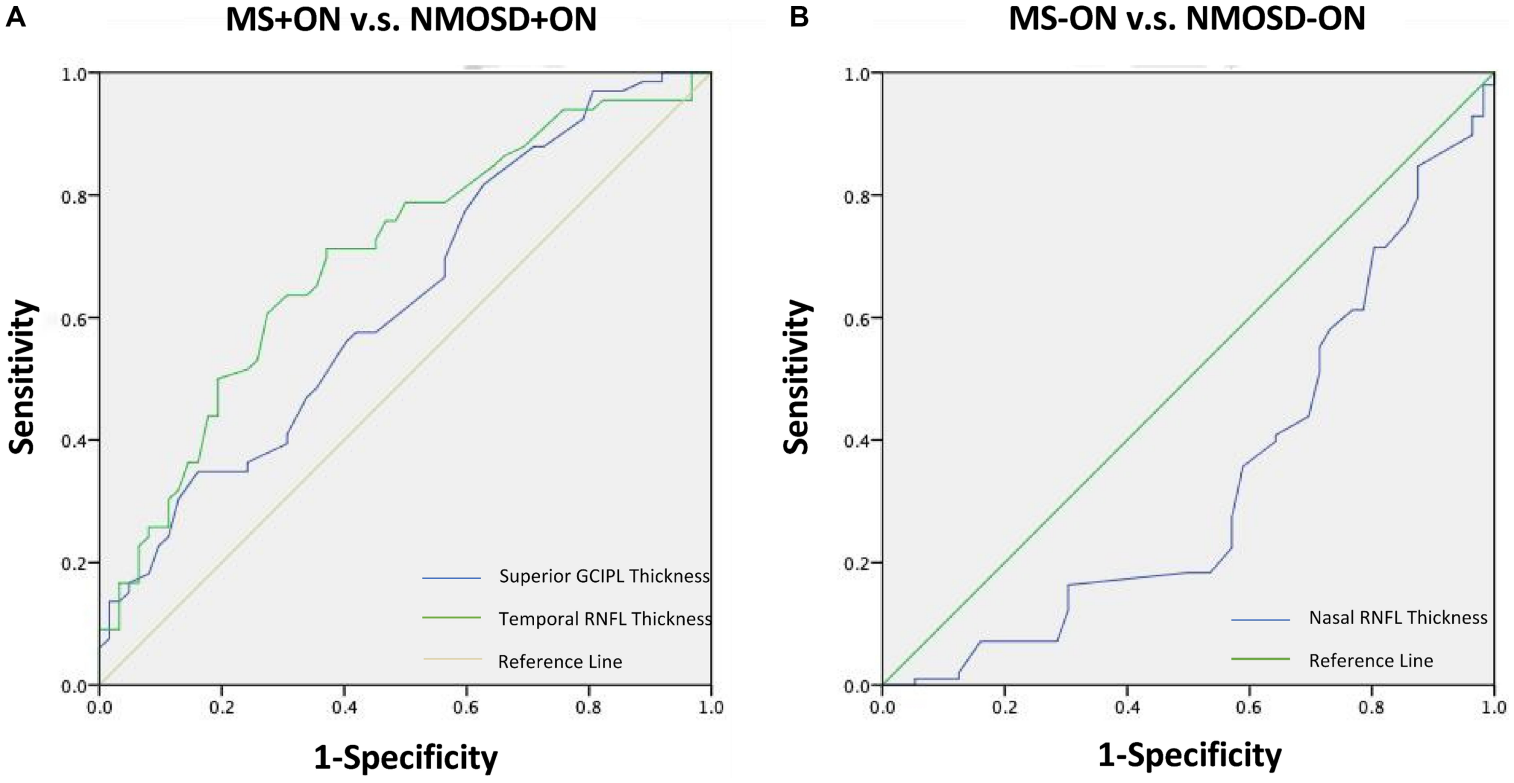
Receiver operating characteristic curves representing the best parameters differentiating NMOSD from MS for ON eyes **(A)** and non-ON eyes **(B)**. MS, Multiple sclerosis; NMOSD, Neuromyelitis optica spectrum disorder; ON, Optic neuritis.

The AUC values of the measurements that differed between the groups are presented in Table 3. The best parameters for differentiating NMOSD eyes from MS eyes were temporal RNFL thickness (AUC, 0.707) and superior GCIPL thickness (AUC, 0.697) for ON eyes (Table 3; Figure 4).

**Table 3:** Diagnostic accuracy of spectral-domain OCT parameters.

	AUC	95%CI	*p* value	Cutoff value	Sensitivity (%)	Specificity (%)
MS + ON v.s. NMOSD+ON						
GCIPL(um)						
Superior	0.697	(0.531–0.753)	0.013	48.5	81.8	37.1
Temporal superior	0.626	(0.530–0.722)	0.014	66.5	36.4	82.3
Temporal inferior	0.625	(0.528–0.721)	0.015	66.5	39.4	82.3
Nasal superior	0.650	(0.497–0.693)	0.064	44.5	95.5	22.6
Nasal temperior	0.649	(0.544–0.735)	0.006	65.5	37.9	83.9
Inferior	0.649	(0.548–0.738)	0.025	51	81.8	38.7
RNFL(um)						
Superior	0.574	(0.472–0.676)	0.149	81.5	89.4	40.3
Temporal	0.707	(0.616–0.788)	0.000	60.5	71.2	62.9
MS-ON v.s. NMOSD-ON						
RNFL(um)						
Nasal	0.337	(0.244–0.430)	0.001	43	98	1.8

NMOSD, neuromyelitis optica spectrum disorder; MS, multiple Sclerosis; ON, optic neuritis; RNFL, etinal nerve fibre layer; GCIPL, ganglion cell-inner plexiform layer.

## Discussion

In this study, in the eyes of patients with +ON, NMOSD decreased more significantly in the superior and temporal regions than did MS, and a history of +ON had no effect on the RNFL thickness of MS patients but made the superior, nasal and temporal thicknesses of NMOSD patients thinner. In the eyes of −ON patients, the RNFL thickness in the MS and NMOSD groups was thinner than that in the control group, and the nasal RNFL thickness was significantly lower in MS patients than in NMOSD patients. This finding is similar to that of previous studies showing a significant reduction in RNFL thickness in both MS and NMOSD patients with optic neuritis (2–5). However, it has also been reported that RNFL thickness is significantly reduced in MS patients without optic neuritis, and there is no significant change in RNFL thickness in NMOSD patients without optic neuritis (6–8). This result is consistent with our results. RNFL thinning reflects damage to unmyelinated ganglion cell axons. These results suggest that the ganglion cell axons in both diseases are damaged to a certain extent. In +ON eyes, the RNFL at the superior and temporior sides is thinner in NMOSD patients than in MS patients, which may indicate that the loss of retinal axons in NMOSD patients is particularly related to a history of optic neuritis. In −ON eyes, damage to the nasal side is more obvious in MS patients than in NMOSD patients. This may be because ON attacks in MS patients are caused mainly by demyelinating lesions, leading to primary retinal neurodegeneration or retrograde transneuronal degeneration, and the axons are relatively well preserved (14). This may also be related to the timing of ON episodes, which may have more severe effects on ganglion cell axons if left untreated, but this needs to be demonstrated by further research. The above findings demonstrated the different patterns of damage in both diseases, with patients with NMOSD exhibiting more axonal loss than patients with MS and demyelination being the predominant change in MS.

In the eyes of patients with +ON, the GCIPL thickness in all regions of NMOSD patients was lower than that in MS patients, and a history of +ON made the GCIPL thickness in all regions of MS patients thinner except for the temporal region, and all regions of NMOSD patients were thinner. In the −ON eye, there was no difference in GCIPL thickness between the two diseases. This finding is consistent with previous studies showing that both the GCL and IPL are thinner in patients with MS and NMOSD and that the GCL and IPL are thinner in patients with NMOSD than in patients with MS (15–17). These findings suggest that patients with NMOSD exhibit more severe neuronal and axonal involvement. Its degeneration is likely to begin with ganglion cell axons (located in the RNFL), progress to the cell body (located in the GCL), and then to dendrites (located in the IPL). The pathogenesis of NMOSD may involve the loss of direct neurons and axons due to the deposition of IgG and complement, whereas the pathogenesis of MS is thought to involve the atrophy of axons and neurons due to the secondary effects of inflammatory demyelination mediated by T-cell activity (18, 19).

In this study, in the -ON eyes, the MS and NMOSD patients had a delayed peak time and decreased amplitude of the P100 wave, and the amplitude of the P100 wave in the +ON eyes was lower than that in the -ON eyes at the 1° and 15′ spatial frequencies in the MS and NMOSD patients. Patients with both diseases have abnormal conduction of optic nerve pathways, and the amplitude of NMOSD decreases more significantly than that of patients with MS does, which is consistent with the findings of previous studies (20–22). Visual evoked potentials (VEPs) are widely used in clinical tests for optic nerve diseases; these tests provide separate measurements of demyelination (peak) and axonal damage (amplitude) in the visual system and are important means of objectively detecting function in the visual system (17). These findings further indicate that patients with NMOSD have more severe axonal loss than patients with MS do.

Moreover, in the analysis of the diagnostic accuracy of the OCT parameters, the temporal RNFL thickness and superior GCIPL thickness of the ON eye were the only parameters that showed differential diagnostic ability between MS and NMOSD (AUCs of 0.707 and 0.697, respectively), which were not found in the −ON eye. These results suggest that the temporal RNFL thickness and superior GCIPL thickness of the ON eye may be important indicators for distinguishing between these two diseases.

Our study had several limitations. First, the age difference in the onset of the two diseases did not match the age, which would have affected the results. Second, the results of brain MRI and AQ4- patients were not included in the analysis, and the mechanism of the difference between the two diseases could not be further analysed. In addition, the cross-sectional nature of the study prevented the analysis of causal inferences. Therefore, confounders need to be removed, the content of the analysis should be increased, and further longitudinal follow-up studies should be conducted to confirm our observations.

## Conclusion

In summary, this study provides a detailed ocular imaging and electrophysiological analysis between NMOSD patients and MS patients and compares the changes in the two diseases with or without ON. The results show that for eyes with ON, NMOSD patients show more severe axonal loss than MS patients do, and the superior, temporal and mean RNFL thicknesses and GCIPL quadrant thicknesses of NMOSD patients are thinner than those of MS patients are. These results suggest that SD-OCT and VEP may be auxiliary diagnostic methods for these two diseases and that abnormalities in RNFL thickness, GCIPL thickness and PVEP may be used to monitor and distinguish the degree of lesions in these two diseases.

## Data Availability

The raw data supporting the conclusions of this article will be made available by the authors, without undue reservation.
